# Molecular characterization of *Blastocystis* sp. in Chinese bamboo rats (*Rhizomys sinensis*)

**DOI:** 10.1051/parasite/2021081

**Published:** 2021-12-14

**Authors:** Junke Song, Xin Yang, Xun Ma, Xuemei Wu, Yuxin Wang, Zhili Li, Guohua Liu, Guanghui Zhao

**Affiliations:** 1 College of Veterinary Medicine, Northwest A&F University Yangling 712100 Shaanxi PR China; 2 College of Life Science and Engineering, Foshan University Foshan 528231 Guangdong PR China; 3 College of Veterinary Medicine, Hunan Agricultural University Changsha 410125 Hunan PR China

**Keywords:** *Blastocystis* sp., Prevalence, Subtyping, *Rhizomys sinensis*, Hunan Province

## Abstract

*Blastocystis* sp., a parasitic eukaryote, widely colonizes the intestines of humans and a large number of animals, including rodents and lagomorphs. More than 30 million bamboo rats (*Rhizomys sinensis*) are farmed in China as a source of meat for human consumption. However, there have been no published articles on *Blastocystis* infection in Chinese bamboo rats prior to the present study. Herein, 480 fresh faecal samples were collected from *R. sinensis* on six farms located in four cities (Wugang, Chenzhou, Huaihua and Jishou) in Hunan Province, south-central China, and were examined for *Blastocystis* infection using polymerase chain reaction (PCR) targeting the small subunit ribosomal RNA (SSU rRNA) gene. The total prevalence of *Blastocystis* in *R. sinensis* was 4.58% (22/480), and significant differences in prevalence were detected among four age groups (<6 months, 6–12 months, 12–24 months and >24 months), with the highest prevalence (7.81%) in rats aged 6–12 months but with no positive samples in rats over 24 months. All farms, except for one in Jishou, were positive for *Blastocystis* infection, with the prevalence ranging from 1.80% to 7.27%. Sequence and phylogenetic analyses revealed two potentially zoonotic subtypes (namely ST4 and ST5) in these rodents, with ST4 predominant in all except one farm in Huaihua. Seven and five sequence types were identified within ST4 and ST5, respectively. This is the first report of *Blastocystis* infection in Chinese bamboo rats and the findings suggest the potential of *R. sinensis* to transmit *Blastocystis* to humans.

## Abstract (fr)

*Blastocystis* sp., un parasite eucaryote, colonise largement les intestins de l’homme et d’un grand nombre d’animaux dont les rongeurs et les lagomorphes. Plus de 30 millions de rats des bambou (*Rhizomys sinensis*) sont élevés en Chine comme source de viande pour la consommation humaine. Cependant, il n’y a eu aucun article publié sur l’infection à *Blastocystis* chez les rats des bambou chinois avant la présente étude. Ici, 480 échantillons de matières fécales fraîches ont été prélevés sur *R. sinensis* dans six fermes situées dans quatre villes (Wugang, Chenzhou, Huaihua et Jishou) de la province du Hunan, dans le centre-sud de la Chine, et ont été examinés pour une infection à *Blastocystis* en utilisant la réaction en chaîne par polymérase (PCR) ciblant le gène de l’ARN ribosomique de la petite sous-unité (ARNr SSU). La prévalence totale de *Blastocystis* chez *R. sinensis* était de 4,58 % (22/480), et des différences significatives de prévalence ont été détectées parmi quatre groupes d’âge (< 6 mois, 6–12 mois, 12–24 mois et > 24 mois), avec la prévalence la plus élevée (7,81 %) chez les rats âgés de 6 à 12 mois mais sans échantillon positif chez les rats de plus de 24 mois. Toutes les fermes, à l’exception d’une à Jishou, étaient positives pour l’infection à *Blastocystis*, avec une prévalence allant de 1,80 % à 7,27 %. Les analyses de séquences et phylogénétiques ont révélé deux sous-types potentiellement zoonotiques (à savoir ST4 et ST5) chez ces rongeurs, ST4 prédominant dans toutes les exploitations sauf une à Huaihua. Sept et cinq types de séquences ont été identifiés parmi ST4 et ST5, respectivement. Il s’agit du premier rapport d’infection à *Blastocystis* chez des rats des bambou chinois et les résultats suggèrent le potentiel de *R. sinensis* à transmettre *Blastocystis* à l’homme.

## Introduction

*Blastocystis* sp., a common parasitic eukaryote, inhabits the intestines of humans and a large number of animals throughout the world [13, 30]. More than one billion people are estimated to be infected with *Blastocystis* globally, and the colonization rate of *Blastocystis* sp. in livestock ranges from 5.5% to 87.1% [6, 7, 13, 26, 28]. However, there are still controversies around the pathogenic potential of *Blastocystis*. Recently, *Blastocystis* sp. was suggested as an indicator for the intestinal health of animals and humans, suggesting a new public health perspective on this protist [5, 9, 15, 18, 27].

The Chinese bamboo rat (*Rhizomys sinensis*) is a species of rodent belonging to the subfamily Rhizomyinae that originally inhabited mountainous areas of Asia, e.g. southern China, Myanmar and Vietnam [24]. Because of good nutritional properties (especially high crude protein, low fat and cholesterol) and medical value, this animal has been farmed since the 1990s as a source of meat in southern China [10]. In 2011, the number of farmed *R. sinensis* was more than 30 million in China, mainly distributed in the Guangxi, Hunan, Guangdong, Jiangxi and Zhejiang Provinces of China [10, 24, 29]. However, due to changes in living conditions and food sources, the resistance of *R. sinensis* to various pathogens is decreasing. In recent decades, several pathogens (e.g. *Escherichia coli*, *Cryptosporidium*, *Penicillium marneffei*, *Giardia duodenalis*, *Trichinella spiralis*) have been detected in *R. sinensis*. Some zoonotic pathogens (e.g. *E. coli*, *P. marneffei*, *G. duodenalis*, *Cryptosporidium* and *T. spiralis*) in these animals also have important implications for human health [3, 11, 12, 29].

Although *Blastocystis* sp. has not been reported in *R. sinensis*, it has been detected in several species of rodents, with a prevalence of 3–100% [8]. Furthermore, of 32 known subtypes, 11 (including potentially zoonotic ST1–ST5, ST7 and ST8, and animal-adapted ST10, ST13, ST15 and ST17) have been reported in captive and wild rodents [8]. In order to understand the infection status of *Blastocystis* sp. in *R. sinensis*, the present study investigated the prevalence and subtypes of *Blastocystis* sp. in *R. sinensis* from Hunan Province and assessed the potentially zoonotic risk of these animals.

## Materials and methods

### Ethics statement

This study was approved by the Research Ethics Committee of Northwest A&F University, Research Ethics Committee of Foshan university, and the Guidance of Laboratory Animal Care and Use of Chinese Ministry of Health, China. All samplings were permitted by farmers and no bamboo rats were hurt during sampling.

### Specimens, DNA extraction and PCR ampliﬁcation

The faecal and DNA samples have already been described [12]. To determine the prevalence of *Blastocystis* sp. in these samples, each genomic DNA sample was amplified by PCR targeting the small sub-unit ribosomal RNA (SSU rRNA) gene with the universal *Blastocystis* primers (BhRDr and RD5) described previously [19]. The PCR products were analysed using 1% agarose gels electrophoresis, and stained with ethidium bromide, and the positive products were sent to Sangon Biotech (Shanghai) Co., Ltd. for direct sequencing in both directions using the PCR primers.

### Sequence and phylogenetic analysis

The sequences obtained were aligned with the reference sequences of *Blastocystis* sp. available in GenBank using the Basic Local Alignment Search Tool (BLAST) from the US National Center for Biotechnology Information (NCBI). All sequences and alignment results were re-checked and manually corrected by eye. Representative sequences for each sequence type were then used for phylogenetic analysis to determine subtypes of *Blastocystis* sp. The neighbour-joining (NJ) method within software MEGA 6.06 [23] was used to construct the genetic tree, with the Kimura 2-parameter model and bootstrap analysis (1000 replications).

### Statistical analysis

A *χ*^2^ test was used to analyse the differences in prevalence among different age groups and regions using SPSS 19.0 software for Windows (SPSS Inc., Chicago, IL, USA). The difference was considered significant when *p* < 0.05.

### Nucleotide sequence accession numbers

Representative nucleotide sequences in the present study have been submitted to GenBank under the accession numbers MK789174–MK789180 and MK789278–MK789282.

## Results and discussion

*Blastocystis* sp. has been reported in rodents in Europe, Asia, Africa and the Americas, including brown rats, house rats, bank vole, chinchilla, yellow necked mouse, wood mouse, gundi, Norway rats, capybara and squirrels, with prevalence ranging from 3% to 100% [8]. However, there are still no data on the occurrence of *Blastocystis* sp. in *R. sinensis*. In the present study, *Blastocystis* infection was investigated in *R. sinensis* for the first time by applying the PCR-sequencing technique based on the SSU rRNA gene. The overall prevalence of *Blastocystis* sp. in *R. sinensis* was 4.58% (22/48), which is higher than in brown rats (3.7%), spontaneous hypertensive rats (3%) and chinchilla (4.2%) from China, and bank vole (3.1%) from the United Kingdom, but is lower than in most rodents reported previously [8]. The differences in prevalence may be due to sampling numbers, detection procedures and different susceptibilities of rodents to *Blastocystis*. For example, only five studies examined over 100 samples of rodents, while no more than 100 or even fewer than 10 faecal samples were examined in other previous studies [8]. Furthermore, the differences in *Blastocystis* prevalence were observed among the six farms from four geographical origins in our study, with the highest prevalence (7.27%) on Farm 3 in Wugang city, and no positive samples were detected on Farm 6 in Jizhou city, but with no significant differences (*χ*^2^ = 4.324, *df* = 5, *p* > 0.05) among these farms (Table 1).

**Table 1:** Factors associated with prevalence of *Blastocystis* infection in *Rhizomys sinensis* in Hunan Province.

Factor	Category		No. examined	No. positive (%)	Subtypes (No.)
Age					
	0–6 months		136	8 (5.88)	ST4 (6), ST5 (2)
	>6–12 months		128	10 (7.81)	ST4 (7), ST5 (3)
	>12–24 months		151	4 (2.65)	ST4 (4)
	>24 months		65	0	0
Location					
	Wugang city	Farm 1	207	12 (5.80)	ST4 (12)
		Farm 2	66	3 (4.55)	ST4 (1), ST5 (2)
		Farm 3	55	4 (7.27)	ST4 (3), ST5 (1)
	Chenzhou city	Farm 4	111	2 (1.80)	ST4 (1), ST5 (1)
	Huaihua city	Farm 5	26	1 (3.85)	ST5 (1)
	Jishou city	Farm 6	15	0	0
Total			480	22 (4.58)	ST4 (17), ST5 (5)

In the present study, significant differences (*χ*^2^ = 7.991, *df* = 3, *p* < 0.05) in *Blastocystis* prevalence were detected in *R. sinensis* among four age groups (<6 months, 6–12 months, 12–24 months and >24 months) (Table 1), with the highest prevalence (7.81%) in rats aged of 6–12 months, but with no findings of any *Blastocystis*-positive faecal sample in rodents over 24 months. Additionally, although the prevalence in *R. sinensis* was slightly lower than that in rats 6–12 months, it seemed that the prevalence deceased with the age. This similar trend was also observed in several previous reports from humans where *Blastocystis* prevalence in children was higher than that in adults, suggesting high susceptibility of young individuals to *Blastocystis* infection [16, 20].

**Figure 1:**
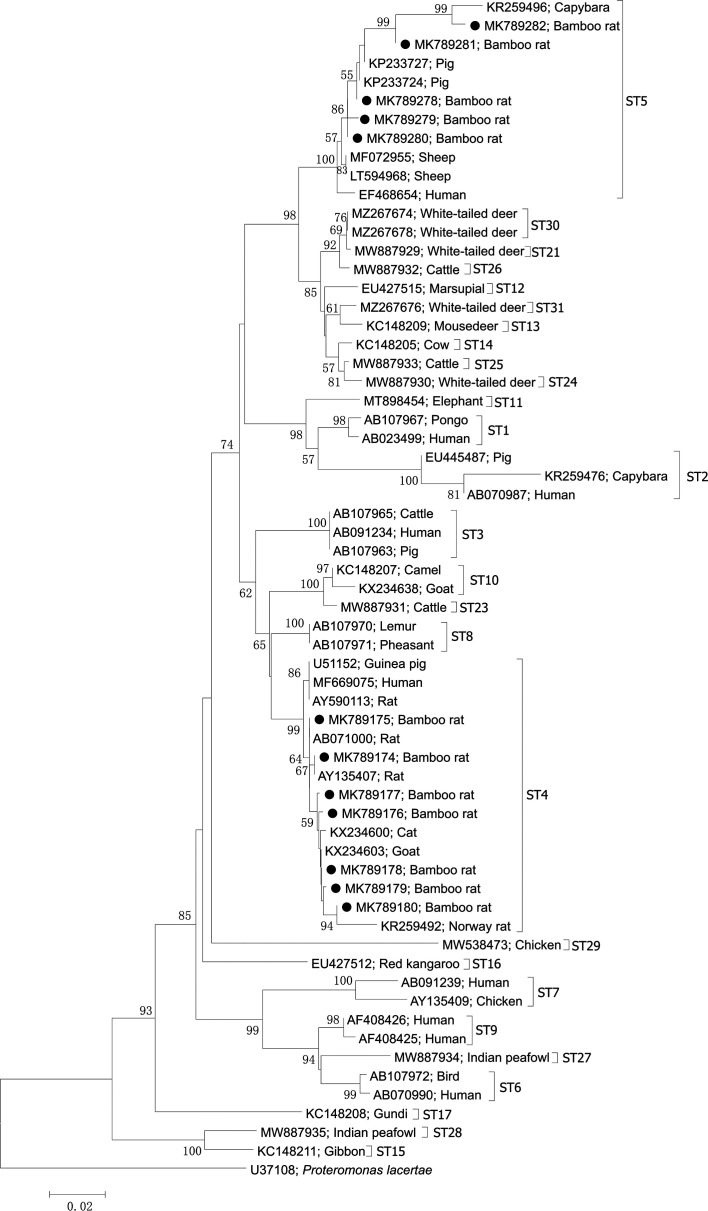
Phylogenetic analysis of *Blastocystis* subtypes in the present study (black filled circles before the accession number) with reference sequence from GenBank based on the SSU rRNA gene fragment by the neighbour-joining method using the Kimura 2-parameter model. Bootstrap values (>50) are indicated at the nodes. Scale bar indicates 0.02 nucleotide substitutions/site. *Proteromonas lacertae* (U37108) is used as the outgroup.

Currently, genetic analysis revealed at least 32 known *Blastocystis* STs in mammals, birds and humans. Sequence and phylogenetic analyses revealed two potentially zoonotic subtypes in *R. sinensis* in the present study (Fig. 1), namely ST4 and ST5. Seven sequence types were identified within ST4, including MK789174 (1), MK789175 (4), MK789176 (1), MK789177 (1), MK789178 (7), MK789179 (2) and MK789180 (1), while five sequence types were found within ST5, including MK789278 (1), MK789279 (1), MK789280 (1), MK789281 (1) and MK789282 (1). ST4 was the predominant subtype found in all positive age groups and sampled farms, except Farm 5 in Huaihua city. Interestingly, ST4 was also identified as being prevalent in other rodents, e.g. brown rats, Norway rats and Polynesian rats [4, 25, 28], suggesting the importance of this subtype for controlling *Blastocystis* infection in rodents. It is notable that two ST4 sequences (MK789177 and MK789178) in the present study were identical to sequences from humans (JN682513 and MH197686) in GenBank, reflecting the potential transmission of ST4 between humans and bamboo rats in this study. Additionally, the ST4 sequences (MK789174, MK789176 and MK789177) in this study were also identical to sequences from other rodents, such as chinchillas (MN124750), Edwards’s long-tailed giant rat (*Leopoldamys edwardsi)* (MT302172) and Chinese hamsters (MN736534) reported in GenBank. ST5 was only found in *R. sinensis* aged less than one year, but it was noted that the ST5 was widely found on four positive farms. This subtype has also been detected in cattle, pigs, chimpanzees, gorillas, gibbons, roe deer, black rhinoceros, goats, camels, ostriches and humans [1, 2, 4, 14, 17, 21, 22, 30]. Interestingly, two ST5 sequences (MK789278 and MK789280) in this study were identical to sequences from pigs (MT373853 and MN493734) in GenBank. Considering the zoonotic potential of these two subtypes, the potential of *R. sinensis* for transmitting *Blastocystis* sp. to humans should be further evaluated.

## Conclusions

The present study revealed the occurrence of *Blastocystis* infection in *R. sinensis*, with an overall prevalence of 4.58%. Phylogenetic analysis identified two potentially zoonotic subtypes in these rodents. This study expanded the host range of *Blastocystis* sp. and provided baseline data for prevention and control of *Blastocystis* sp. in *R. sinensis*.

## Conflicts of interest

The authors declare that they have no conflict of interest.
